# OsNRAMP5 contributes to manganese translocation and distribution in rice shoots

**DOI:** 10.1093/jxb/eru259

**Published:** 2014-06-24

**Authors:** Meng Yang, Yuanyuan Zhang, Lejing Zhang, Jintao Hu, Xing Zhang, Kai Lu, Huaxia Dong, Dujun Wang, Fang-Jie Zhao, Chao-Feng Huang, Xingming Lian

**Affiliations:** ^1^National Key Laboratory of Crop Genetic Improvement and National Center of Plant Gene Research (Wuhan), Huazhong Agricultural University, Wuhan 430070, China; ^2^State Key Laboratory of Crop Genetics and Germplasm Enhancement, College of Resources and Environmental Science, Nanjing Agricultural University, Nanjing 210095, China

**Keywords:** Cadmium, manganese, *OsNRAMP5*, rice, root-to-shoot translocation, transporter.

## Abstract

Manganese (Mn) is an essential micronutrient for plants playing an important role in many physiological functions. OsNRAMP5 is a major transporter responsible for Mn and cadmium uptake in rice, but whether it is involved in the root-to-shoot translocation and distribution of these metals is unknown. In this work, *OsNRAMP5* was found to be highly expressed in hulls. It was also expressed in leaves but the expression level decreased with leaf age. High-magnification observations revealed that OsNRAMP5 was enriched in the vascular bundles of roots and shoots especially in the parenchyma cells surrounding the xylem. The *osnramp5* mutant accumulated significantly less Mn in shoots than the wild-type plants even at high levels of Mn supply. Furthermore, a high supply of Mn could compensate for the loss in the root uptake ability in the mutant, but not in the root-to-shoot translocation of Mn, suggesting that the absence of OsNRAMP5 reduces the transport of Mn from roots to shoots. The results suggest that OsNRAMP5 plays an important role in the translocation and distribution of Mn in rice plants in addition to its role in Mn uptake.

## Summary

OsNRAMP5 plays an important role in the translocation and distribution of Mn in rice plants in addition to its role in Mn uptake.

## Introduction

Manganese (Mn), an essential micronutrient for most organisms, is involved in many biochemical pathways as an enzyme cofactor. Mn deficiency in plants can affect the biosynthesis of fructans and structural carbohydrates, causing leaves to become slack and soft (Marschner, 1995). In addition, Mn deficient plants are more susceptible to low-temperature stress and pathogen infection, resulting in lower crop yields (Hebbern *et al.*, 2005; Marschner, 1995). However, when accumulated in excess, Mn can have various phytotoxic effects, including reduction of growth, photosynthesis and chlorophyll content, inhibition of enzyme activities, and damage to chloroplasts (Doncheva *et al.*, 2005; Keen *et al.*, 2000; Mora *et al.*, 2009).

Recently, great progress has been made concerning the mechanism of Mn uptake in plants. A number of genes responsible for the uptake of Mn by roots have been identified (Cailliatte *et al.*, 2010; Pedas *et al.*, 2008). In barley (*Hordeum vulgare*), two separate Mn transport systems that mediate high-affinity and low-affinity Mn^2+^ influx at different concentrations were identified (Pedas *et al.*, 2005). Further studies on Mn uptake in barley showed that HvIRT1, targeted to the plasma membrane, plays an important role in controlling Mn^2+^ uptake in roots. Heterologous expression of *HvIRT1* in the Δ*smf1* yeast mutant, which is deficient in Mn^2+^ uptake, restored its growth. In addition, a comparison of plants with differing ability to grow in soils with low Mn^2+^ showed that the expression of HvIRT1 was 40% greater in plants with an Mn-efficient genotype compared with plants with an Mn-inefficient genotype, and the higher expression of HvIRT1 was correlated with an increased Mn2+ uptake rate (Pedas *et al.*, 2008). *AtNRAMP1*, a member of the gene family natural resistance associated macrophage protein (NRAMP) in *Arabidopsis*, was recently reported to be essential for Mn^2+^ uptake under Mn-deficient conditions (Cailliatte *et al.*, 2010). AtNRAMP1 encodes a plasma membrane protein in the root cells and mediates a high-affinity transporter of Mn^2+^ with an apparent *K*m value of 28nM. Growth of the knockout mutant of *AtNRAMP1* was inhibited under Mn-deficient conditions much more than the wild type.

Analysis of the tolerance mechanisms to Mn excess in plants is another focus of current research and has contributed substantially to the present understanding of intracellular Mn compartmentation (Pittman, 2005). Vacuoles play a very important role in Mn tolerance in plants. Expression of *AtCAX2* in tobacco enables more Mn to accumulate in vacuoles and increases Mn tolerance compared with wild type (Hirschi *et al.*, 2000). ShMTP1, a member of the cation diffusion facilitator (CDF) family from *Stylosanthes hamata*, conferred Mn^2+^ tolerance in yeast by internal sequestration rather than by efflux of Mn^2+^. When expressed in *Arabidopsis*, ShMTP1 is localized to the tonoplast and can also confer Mn tolerance by sequestering Mn^2+^ in this organelle (Delhaize, 2003). Another CDF member, AtMTP11, might be responsible for a secretory pathway-mediated mechanism in Mn detoxification in plants. AtMTP11 was targeted to a Golgi-like compartment, and *AtMTP11*-overexpressing plants were hyper-tolerant to Mn (Delhaize *et al.*, 2007; Peiter *et al.*, 2007).

A number of transporters have been found to be involved in Mn transport in rice (*Oryza sativa* L.). OsYSL2, a member of the yellow stripe 1-like (YSL) proteins, has a Mn^2+^ transport activity and contributes to the long-distance transport of Mn (Ishimaru *et al.*, 2010; Koike *et al.*, 2004). OsYSL6 is another member of the YSL family that has been shown to be a Mn-nicotianamine (NA) transporter required for the detoxification of high levels of Mn (Sasaki *et al.*, 2011). Furthermore, OsNRAMP3 is able to transport Mn^2+^ and is specifically expressed in the vascular bundles, playing a role in the distribution of Mn between young and old tissues (Yamaji *et al.*, 2013; Yang *et al.*, 2013).

Recently, another member of the NRAMP family in rice, OsNRAMP5, was reported to be a Mn and Cd transporter involved in the root uptake of these metals from the medium (Ishikawa *et al.*, 2012; Ishimaru *et al.*, 2012a; Ishimaru *et al.*, 2012b; Sasaki *et al.*, 2012). Ishimaru *et al.* (2012b) found that OsNRAMP5 complemented the growth of yeast *Δsmf1*, whereas knockdown of OsNRAMP5 decreased Mn accumulation in both roots and shoots of rice plants. However, whether OsNRAMP5 participates in the distribution of Mn from roots and shoot and in the shoot tissues remains unknown. In the present study, the role of OsNRAMP5 in Mn distribution in rice plants was investigated.

## Materials and methods

### Plant materials

Wild-type, mutant and transgenic plants used in the present study were all based on the background of the cv. Zhonghua 11 (*O. sativa* L. ssp. *japonica* variety). The *osnramp5* mutant is a T-DNA insertion mutant identified from the Rice Mutant Database (Wu *et al.*, 2003; Zhang *et al.*, 2006).

### Hydroponic experiments

Hydroponic experiments were performed using a standard rice culture solution (1.44mM NH_4_NO_3_, 0.3mM NaH_2_PO_4_, 0.5mM K_2_SO_4_, 1.0mM CaCl_2_, 1.6mM MgSO_4_, 0.17mM Na_2_SiO_3_, 50 µM Fe-EDTA, 0.06 µM (NH_4_)_6_Mo_7_O_24_, 15 µM H_3_BO_3_, 8 µM MnCl_2_, 0.12 µM CuSO_4_, 0.12 µM ZnSO_4_, 29 µM FeCl_3_, 40.5 µM citric acid, pH 5.5) described by Yoshida *et al.* (1976). In the Mn concentration gradient experiments, a range of Mn concentrations (0.04, 0.08, 0.2, 0.4, 0.8, 1.6, 8, 80, and 800 µM) were imposed, with the 8 µM Mn treatment as the control. In some experiments 2nM of CdCl_2_ was added to the hydroponic solution to investigate Cd uptake.

### Promoter fusion and RNA in situ hybridization

To create the *OsNRAMP5*-promoter:*GUS* construct, 2.06kb of the genomic sequence located upstream of the *OsNRAMP5* initiation codon was amplified by PCR from Zhonghua 11 genomic DNA using primers Pnr5-F (5’-agggatcccgcaactcccacaactactg-3’) and Pnr5-R (5’-tcggatccgcttcctctcttagcttcttca-3’). The amplified promoter fragment was digested by *Bam*H1 and introduced into the vector pDX2181 in the correct direction (Ye *et al.*, 2012). Wild-type Zhonghua 11 calli was transformed with this construct. The transgenic plant tissues were incubated in a X-Gluc staining buffer at 37 °C for 4h (Jefferson *et al.*, 1987).

Hybridization and immunological detection were performed as described in Xue *et al.* (2008). The probe of *OsNRAMP5* was amplified from Zhonghua 11 by PCR using gene-specific primers *in situ*-F (5’-cgacgagcccttgccgta-3’) and *in situ*-R (5’-tctgcgagcgatctggacc-3’) and cloned into the pGEM-T vector (Promega). The sense and antisense probes were transcribed *in vitro* by T7 or SP6 transcriptase using a Digoxigenin RNA labelling kit (Roche).

### RNA extraction and real-time PCR

Total RNA was extracted using Trizol reagent (Invitrogen). The first-strand cDNAs were synthesized with 3 µg of total RNA in 20 µl of reaction mixture using SuperScript III reverse transcriptase (Invitrogen) according to the manufacturer’s instructions. Real-time PCR was performed using the SYBR Premix Ex Taq^TM^ (TaKaRa) with the following gene-specific primers: rqNR5-F (5’-cgctcccaaggtagagaagaagaa-3’) and rqNR-R (5’-atataaacgaacggctccgacgca-3’). The rice *Ubiquitin5* gene was used as the internal control with following primers: qUbq-F (5’-aaccagctgaggcccaaga-3’) and qUbq-R (5’-acgattgatttaaccagtccatga-3’).

### Elemental analysis

Shoots and roots were harvested separately and roots were washed with distilled water twice before sampling. All samples were dried at 80 °C for 3 d and then digested in 65% nitric acid in a MARS6 microwave (CEM) with a gradient of temperatures from 120 °C to 180 °C for 45min. After dilution in deionized water, the metal contents of the samples were determined by inductively coupled plasma mass spectrometry (ICP-MS, Agilent 7700 series, USA).

## Results

### The expression profile of *OsNRAMP5* in different tissues of rice

**Fig. 1.**
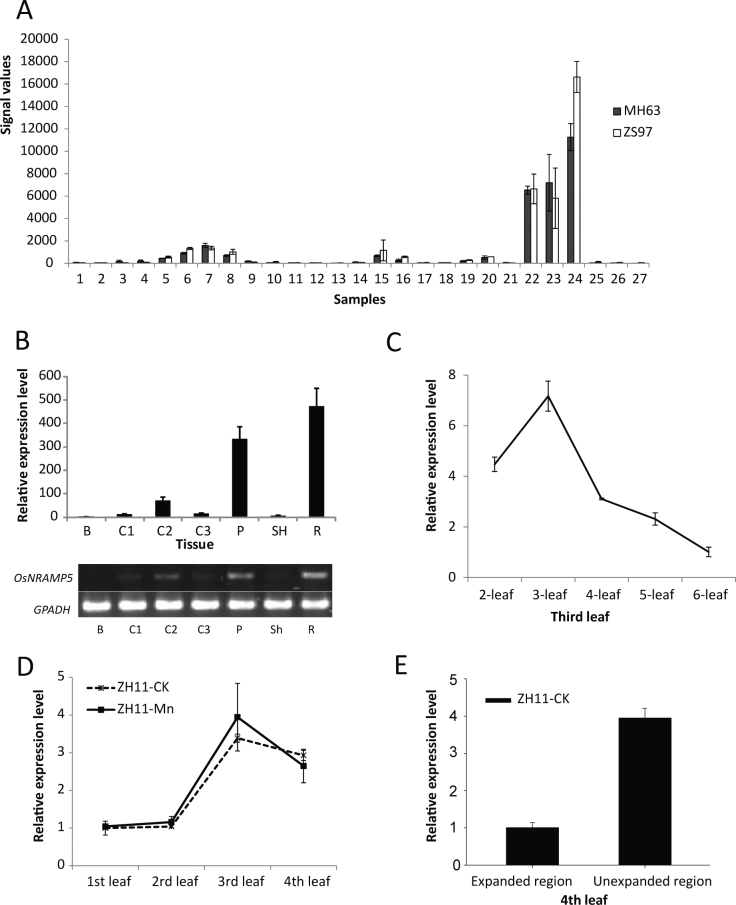
The transcript levels of *OsNRAMP5* in different tissues of rice plants. (A) The transcript levels of *OsNRAMP5* in 27 samples representing different organs or tissues at different developmental stages of the Minghui 63 (MH63) and Zhenshan 97 (ZS97) based on signal values from Wang *et al.* (2010). Numbers from 1 to 27 represent the following 27 samples: (1) calli, 15 days after subculture; (2) calli, screening stage; (3) calli, 5 days after regeneration; (4) seed, 72h after imbibition; (5) embryo and radicle after germination; (6) leaf and root at three-leaf stage; (7) root, seedling with 2 tillers; (8) shoot, seedling with 2 tillers; (9) sheath, young panicle at stage 3; (10) leaf, young panicle at stage 3; (11) leaf, 4–5cm young panicle; (12) flag leaf, 5 days before heading; (13) flag leaf, 14 days after heading; (14) sheath, 4–5cm young panicle; (15) stem, 5 days before heading; (16) stem, heading stage; (17) young panicle at stage 3; (18) young panicle at stage 4; (19) young panicle at stage 5; (20) panicle, 4–5cm young panicle; (21) stamen, one day before flowering; (22) panicle, heading stage; (23) spikelet, 3 days after pollination; (24) hull, one day before flowering; (25) endosperm, 7 days after pollination; (26) endosperm, 14 days after pollination; (27) endosperm, 21 days after pollination. (B) Quantitative RT-PCR analysis of the expression pattern of *OsNRAMP5* in various tissues from Zhonghua 11 (ZH11). B, leaf blade; C1, culm I; C2, culm II; C3, culm III; P, panicle; Sh, sheath; R, root. (C) Quantitative RT-PCR analysis of the *OsNRAMP5* transcriptional level in the third leaf of Zhonghua 11 seedling at the two-to-six-leaf stages. (D) The expression level of *OsNRAMP5* in different leaves of rice plants at different Mn supplies; the 1st leaf is the oldest of the four leaves from the tiller and the 4th leaf is not fully expanded. (E) The expression pattern of *OsNRAMP5* in different regions of the 4th leaf. Data are means ± SD of three biological replicates in real-time PCR.

The expression pattern of *OsNRAMP5* in different tissues of rice was first extracted from the CREP database (http://crep.ncpgr.cn; Wang *et al.*, 2010); the microarray data have been verified by qPCR to reveal the expression profiles of various genes (Huang *et al.*, 2009; Ma *et al.*, 2009; Nuruzzaman *et al.*, 2008). The results from the database for 27 tissues of cv. Minghui 63 (*O. sativa* L. ssp*. indica*) and cv. Zhenshan 97 (*O. sativa* L. ssp*. indica*), each with two replications, indicated that *OsNRAMP5* was expressed in rice with a strong tissue-specific pattern (Fig. 1A). *OsNRAMP5* exhibited the strongest expression signal in young reproductive tissues, e.g. panicle, spikelet, and hull. *OsNRAMP5* also showed high expression levels in plumules and young shoots, leaves and roots, and a moderate level in stems at the heading stage. In contrast, little or no expression of *OsNRAMP5* was found in the mature leaf, flag leaf, leaf sheath, or endosperm.

To confirm the expression pattern, the specific accumulation of *OsNRAMP5* mRNA was determined in seven different tissues of cv. Zhonghua 11 by RT-PCR and real-time PCR (Fig. 1B). *OsNRAMP5* was highly expressed in roots and panicles, moderately in culms, but very little in leaf blades and sheaths. This pattern is highly consistent with the microarray data described above. To further understand the *OsNRAMP5* expression pattern in leaves, the *OsNRAMP5* transcript levels were monitored in the third leaf of wild-type seedlings during the growth stage from two to six leaf. At the two-leaf stage, the third leaf was wrapped inside the second leaf sheath and was not fully developed. At the three-leaf stage, the third leaf had fully expanded, and by the six-leaf stage, the third leaf had senesced slightly. Real-time RT-PCR showed relatively high transcript levels of *OsNRAMP5* in the third leaf at the young stage, which then decreased gradually (Fig. 1C). The expression of *OsNRAMP5* was also compared among different leaves of young wild-type plants. The expression of *OsNRAMP5* was higher in younger than in older leaves under both Mn-sufficient and Mn-deficient conditions (Fig. 1D). Furthermore, the expression of *OsNRAMP5* was clearly higher in the unexpanded than in the expanded part of the fourth leaf (Fig. 1E).

It was reported that the expression of the high-affinity Mn transporter AtNRAMP1 in *Arabidopsis* roots was induced by Mn deficiency (Cailliatte *et al.*, 2010). Similarly, many genes involved in Fe uptake also show enhanced expression under Fe-limited conditions (Koike *et al.*, 2004). Time-course experiments were conducted to test whether the expression of *OsNRAMP5* was affected by Mn or Fe deficiency. Real-time RT-PCR showed that the transcript level of *OsNRAMP5* in roots was not obviously affected by the absence of Fe or Mn in the hydroponic solution (Supplementary Fig. S1 available at *JXB* online), which is consistent with the result described by Ishimaru *et al.* (2012b). By contrast, the expression of *OsNRAMP5* in shoots was induced by Fe deficiency after 9 days in the –Fe treatment and the induction was further increased on day 13. By contrast, the expression of *OsNRAMP5* was not affected by the deficiency of Mn (Supplementary Fig. S1 available at *JXB* online).

### The expression pattern of *OsNRAMP5* at the cellular level

**Fig. 2.**
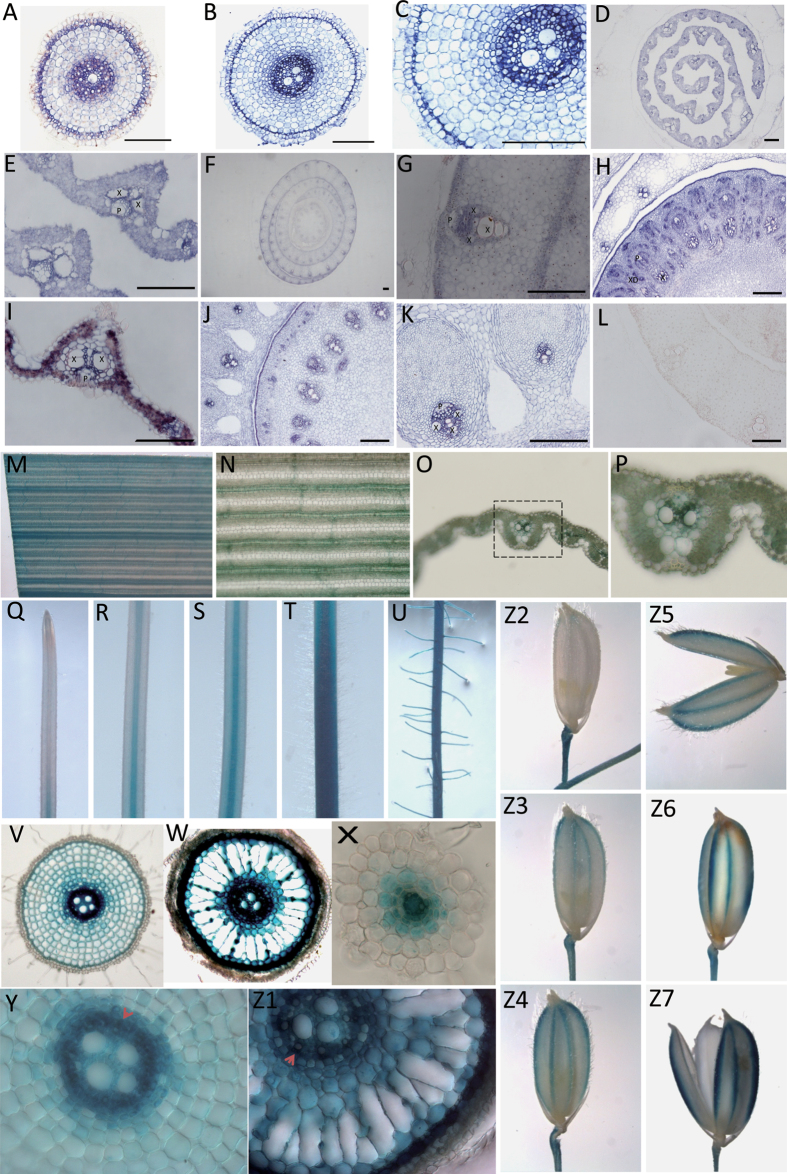
The expression pattern of *OsNRAMP5*. (A–L) *In situ* hybridization of *OsNRAMP5*. (A–C) Transverse section of roots. (A) The elongation zone of root. (B) The mature zone of root. (C) Magnified image of part of B. (D) Transverse section of unexpanded leaf. (E) Magnified image of part of D. (F) Transverse section of a young tiller. (G) Magnified image of part of F. (H) Transverse section of culm. (I) Transverse section of expanded leaf. (J) Transverse section of the border region of the node. (K) Magnified image of part of J. (L) A negative control detected by the sense probe. (M–Z7) Expression pattern of *OsNRAMP5* detected in plants transformed with the construct *OsNRAMP5*-promoter:GUS by histochemical straining of GUS activity. (M–Z1) The localization of *OsNRAMP5* in leaves and roots of a plant cultivated hydroponically for two weeks. (M) The expanded young leaf. (N) Magnified image of part of M. (O) Transverse section of leaf shown in M. (P) Enlarged view of a vascular bundle from O. (Q) The tip of root. (R) The elongation zone of root. (S, T) The basal (S) and the mid (T) mature zone of root. (U) The lateral roots. (V) The transverse section of R. (W) The transverse section of T. (X) The transverse section of a small lateral root described in U. (Y) Magnified image of part of V. (Z1) Magnified image of part of W. (Z2–Z7) The localization of *OsNRAMP5* in hulls. (Z2) The hull, 5 days before heading. (Z3) The hull, 2 days before heading. (Z4, Z5) The hull at heading stage. (Z6, Z7) The hull and endosperm, 21 days after pollination. X, xylem region of regular vascular bundles; XD, xylem region of diffuse vascular bundles; P, phloem region of regular vascular bundles. Scale bars (A–L) =100 µm.

To study the cellular localization of *OsNRAMP5* expression, RNA *in situ* hybridization was performed in roots, unexpanded leaves, young tillers, culms, expanded leaves, and the border region of nodes (Fig. 2A–L). The intensity of signals showed that *OsNRAMP5* was highly expressed in the stele and sclerenchyma layer of roots, unexpanded leaves, culms, and the border region of nodes, but only moderately expressed in expanded leaves. There was little expression in the mature leaf sheaths. Furthermore, high-magnification observation showed an enrichment of *OsNRAMP5* expression in the vascular bundles, especially in the parenchyma cells surrounding the xylem (Fig. 2E, G, I, K).

To complement the *in situ* hybridization results, a 2063-bp promoter region of *OsNRAMP5* was used to direct beta-glucuronidase (*GUS*) expression in transgenic rice. GUS staining showed a strong expression of *OsNRAMP5* in the leaf vascular bundles (Fig. 2M–P), consistent with the pattern revealed by *in situ* hybridization. In roots, the GUS activity was mainly detected in the elongation and maturation zones and in lateral roots, but not in root caps and the meristem zone (Fig. 2Q–U). Transverse sections of roots showed that the stele and sclerenchyma layer cells had the highest GUS activity (Fig. 2V–X), which confirmed the *in situ* hybridization results. Furthermore, high-magnification observation of transverse sections of roots identified the highest accumulation of *OsNRAMP5* transcripts in the xylem of both the elongation and maturation zones (Fig. 2Y, Z1). In the maturation region of root, GUS activity was found in all cell types, including exodermis and endodermis. GUS activity was also detected in the vascular tissues of hulls from 2 d before heading to the stage of grain ripening (Fig. 2Z2–Z6). However, no GUS activity was detected in the endosperm (Fig. 2Z7).

### Knockout of *OsNRAMP5* increased the sensitivity to Mn and Fe deficiency

**Fig. 3.**
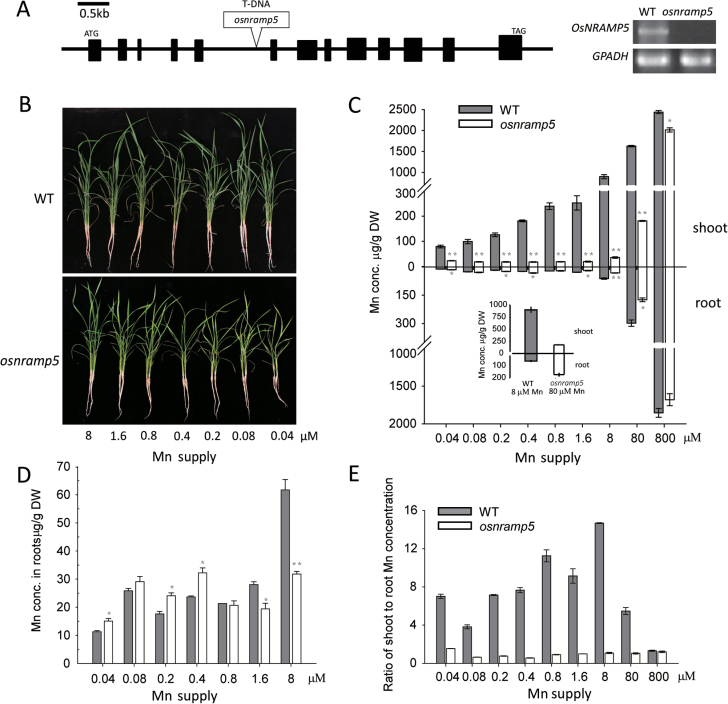
Identification of a knock-out line of *OsNRAMP5*. (A) The structure of *OsNRAMP5*. 13 exons (boxes) and 12 intron (lines between boxes) are contained in *OsNRAMP5* and a T-DNA inserted in the 5th intron of the *osnramp5* plant results in a complete loss of *OsNRAMP5* transcriptional level as shown by RT-PCR. The growth phenotype (B), Mn concentrations (C), and the ratio of shoot to root Mn concentrations (E) were analysed at different Mn supplies. The Mn concentrations of wild type plants at 8 µM Mn and mutant plants at 80 µM Mn were extracted from C and are shown in C insert. (D) Enlarged view of root Mn concentrations from (C). Data are means ± SD of three biological replicates, and five plants were mixed in one replication for metal determination. One and two asterisks indicate values are significantly different from the wild type (WT) at the levels of *P*<0.05 and *P*<0.01, respectively (*t*-test).

To further investigate the biological function of OsNRAMP5, a T-DNA insertion mutant, named *osnramp5* from RMD (Rice Mutant Database: http://rmd.ncpgr.cn; Zhang *et al.*, 2006), was identified. In this mutant, the T-DNA was inserted in the fifth intron of *OsNRAMP5* (Fig. 3A), different from the allelic *nramp5* mutant described by Sasaki *et al.* (2012). Transcripts of *OsNRAMP5* were not detectable in the *osnramp5* mutant by RT-PCR, indicating that the gene has been knocked out. When supplied with a sufficient level of Mn (8 µM), *osnramp5* showed a similar phenotype to wild-type plants (Supplementary Fig. S2 available at *JXB* online). At lower levels of Mn supply (0.04–1.6 µM), the *osnramp5* mutant plants showed reduced growth compared with the wild-type plants, suggesting that the mutant was more sensitive to Mn deficiency. The mutant was also slightly more sensitive to Fe deficiency, but not to the deficiencies of potassium (K), calcium (Ca), copper (Cu), magnesium (Mg), or zinc (Zn) (Supplementary Fig. S2 available at *JXB* online). These phenotypic differences were further supported by the concentrations of the metals in plants. Mn concentration was much lower in both roots and shoots of *osnramp5* compared with wild-type plants, especially in the shoots in which Mn concentration was only about 4% of that in the wild-type plants (Supplementary Fig. S3 available at *JXB* online). By contrast, there were no significant differences in the concentrations of K, Ca, Mg, Cu, or Zn in either the control or the nutrient-deficient treatments (Supplementary Fig. S3 available at *JXB* online).

To identify the role of OsNRAMP5 in Mn uptake and translocation in rice, a Mn concentration gradient experiment was performed. Plants were initially grown with the normal nutrient solution containing 8 µM Mn for 12 d and then transferred to treatments containing 0.04–800 µM Mn for another 18 d. Whereas wild-type plants grew normally in all treatments, growth of the *osnramp5* plants decreased obviously when Mn concentration in the nutrient solution was lowered to 8 µM or less (Fig. 3B). In these treatments typical symptoms of Mn deficiency (stunted growth and leaf yellowing) were observed only in the *osnramp5* plants. Moreover, shoot Mn concentrations of the *osnramp5* plants were markedly lower than those of wild-type (3.5–25-fold difference; Fig. 3B, C), indicating that Mn deficiency was the cause of the growth reduction in the mutant. However, the response of root Mn concentration was different. In the low Mn treatments (0.04–0.8 µM), *osnramp5* roots had either similar or significantly higher concentration of Mn than wild-type roots (Fig. 3D). Only in the sufficient-high Mn treatments (1.6–80 µM) did *osnramp5* accumulate lower concentrations of Mn in the roots than wild-type (Fig. 3C). *osnramp5* roots accumulated a much higher Mn concentration in the 80 µM Mn treatment than did wild-type plants grown under 8 µM Mn supply, but this did not rescue the shoot Mn concentration to the level of wild-type plants grown with 8 µM Mn (Fig. 3C insert). The ratios of shoot-to-root Mn concentrations were significantly higher, by 4.7–13.1 times, in wild-type than *osnramp5* in all Mn treatments except the highest level (800 µM) (Fig. 3E). These results suggest that that OsNRAMP5 not only contributes to Mn uptake but also to the root-to-shoot translocation of Mn.

### Uptake of Fe and Cd mediated by OsNRAMP5 was affected by Mn concentration

**Fig. 4.**
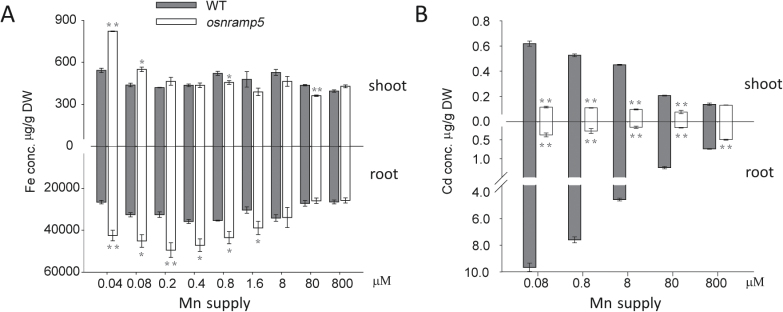
The concentrations of Fe and Cd at different Mn supplies. The concentrations of Fe (A) and Cd (B) of wild-type and *osnramp5* mutant plants were analysed at different Mn supplies. Data are means±SD of three biological replicates, and five plants were mixed in one replication for metal determination. One and two asterisks indicate values are significantly different from the WT at the levels of *P*<0.05 and *P*<0.01, respectively (*t*-test).

The NRAMP family transporters in *Arabidopsis* have been shown to transport Fe, Mn, and Cd (Lanquar *et al.*, 2004; Lanquar *et al.*, 2010; Thomine *et al.*, 2003). To investigate the role of OsNRAMP5 in Fe and Cd transport, Fe and Cd concentrations of *osnramp5* and wild-type plants were determined under different Mn supplies. The concentration of Fe in the roots of *osnramp5* was significantly higher than that of wild type when plants were grown at low Mn concentrations (≤1.6 µM); at high Mn concentrations there were no significant differences (Fig. 4A). In shoots, a significantly higher Fe concentration was found in the mutant only in the two lowest Mn treatments. Compared with wild type, *osnramp5* plants contained significantly lower Cd concentrations in both roots and shoots (Fig. 4B). Increasing the concentration of Mn in the nutrient solution markedly decreased the accumulation of Cd in both roots and shoots of wild type, but not in *osnramp5* plants. As a result, the difference between wild-type and *osnramp5* in root and shoot Cd concentrations decreased from 26 and 5.4 times, respectively, in the 0.04 µM Mn treatment to only 1.5 and 1.1 times in the 800 µM Mn treatment.

Because Fe and Cd accumulations were affected by different Mn supply, the expression of some other Fe and Cd transporters in rice root were investigated. The expression levels of *OsIRT2*, *OsNRAMP1*, *OsYSL2*, and *OsYSL15* were suppressed by low Mn conditions, but the expression levels of *OsIRT1*, *OsYSL16*, and *OsYSL18* was unaffected (Supplementary Fig. 4 available at *JXB* online). The results were consistent with those of rice plants grown under Mn deficiency (Supplementary Fig. 5 available at *JXB* online). By contrast, the expression levels of *OsIRT2*, *OsNRAMP1*, *OsYSL2*, and *OsYSL15* were highly induced by Fe deficiency. Moreover, much higher levels of expression of these four genes were observed in *osnramp5* mutant than wild type (Supplementary Fig. 6 available at *JXB* online).

**Fig. 5.**
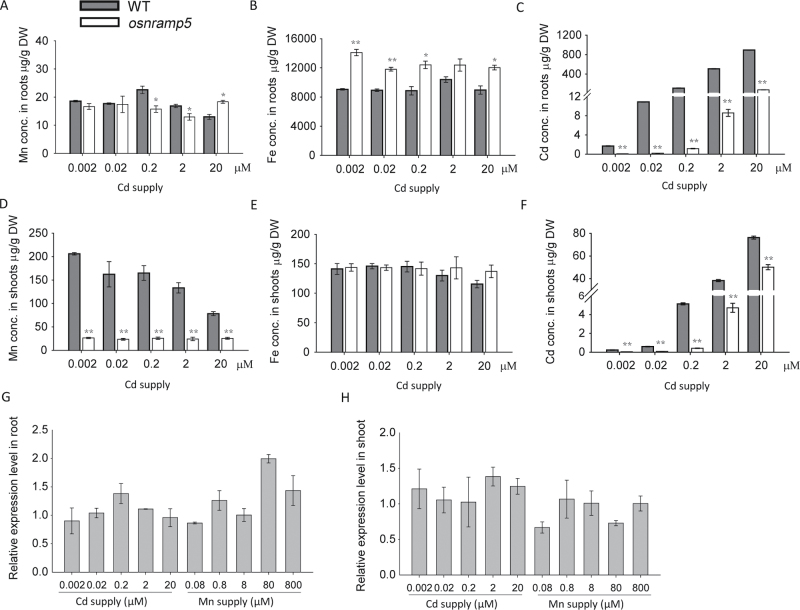
The metal concentrations and expression levels at different Cd supplies. Plants were grown under normal condition for two weeks and then transferred to different Cd supplies for additional two weeks. Mn, Fe, and Cd concentrations were analysed in roots (A–C) or shoots (D–F) of rice plants at different Cd supplies. Expression levels of *OsNRAMP5* of roots and shoots at different Mn or Cd supplies were analysed in (G) and (H) respectively. Data are means±SD of three biological replicates, and five plants were mixed in one replication for metal determination. One and two asterisks indicate values are significantly different from the WT at the levels of *P*<0.05 and *P*<0.01, respectively (*t*-test).

To investigate whether Mn accumulation was affected by different Cd supply, a Cd concentration gradient experiment was conducted (Fig. 5). Wild-type and *osnramp5* plants were grown under normal conditions for two weeks and then transferred to normal nutrient solution with different Cd concentrations for another two weeks. As expected, the Cd concentrations of roots and shoots in both wild-type and mutant plants increased with Cd supply (Fig. 5C, F). However, *osnramp5* plants accumulated much less Cd in both roots and shoots than wild-type plants, representing only 1.0–8.6% for root and 8.2–65.7% for shoot of the wild-type’s Cd concentrations. Cd treatment had no significant effect on Mn concentration in roots (Fig. 5A), but decreased Mn concentration in shoots of wild-type plants. This antagonistic effect was not seen in the mutant (Fig. 5D). No such suppression was observed for Fe concentrations (Fig. 5B, E).

### Fe deficiency affected Mn accumulation in both knockout and wild-type lines

**Fig. 6.**
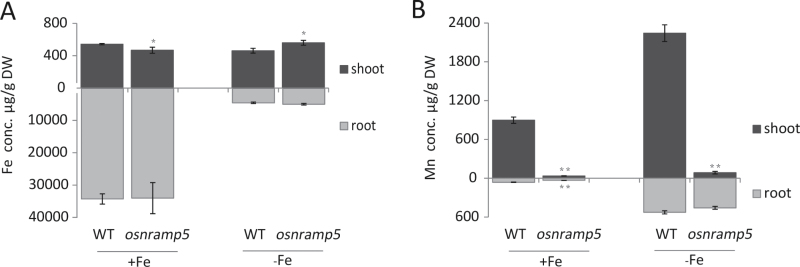
The concentrations of Mn and Fe in response to Fe deficiency. Plants were grown under normal condition for 12 d and then transferred to Fe-free (–Fe) medium or continued Fe-replete (+Fe) medium for additional 18 d. The Fe (A) and Mn (B) concentrations were determined in both roots and shoots. Data are means ± SD of three biological replicates, and five plants were mixed in one replication for metal determination. One and two asterisks indicate values are significantly different from the WT at the levels of *P*<0.05 and *P*<0.01, respectively (*t*-test).

*Osnramp5* and wild-type plants were cultivated for 12 d under normal conditions and then transferred to Fe-deficient (–Fe) or normal (+Fe) nutrient solution for an additional 18 d. *Osnramp5* plants seemed more sensitive to Fe deficiency than wild type (Supplementary Fig. S2 available at *JXB* online). Fe concentrations in roots were markedly lower in the –Fe treatment but there was no significant difference between the mutant and wild-type plants (Fig. 6A). Compared with plants grown under normal conditions, both the mutant and wild-type plants grown in –Fe solution accumulated almost tenfold more Mn in roots (Fig. 6B). In addition, Fe-deficient conditions narrowed the difference in the root Mn concentration between the mutant and wild-type plants; the *osnramp5* plants accumulated 87% of the Mn level of wild-type in the –Fe treatment but only 52% in the +Fe treatment. The shoots showed a similar tendency under Fe-deficient conditions, in which Mn concentration increased in both mutant and wild-type plants (Fig. 6B). Despite the large increase in Mn concentration in *osnramp5* roots under Fe-deficient conditions, the effect on shoot Mn concentration was very small (Fig. 6B). In this treatment shoot Mn concentration of *osnramp5* was only 4% of that in the wild type, suggesting that *osnramp5* plants lost the ability to transport Mn from roots to shoots.

### Mn and Cd showed different distributions in leaves and were affected by the absence of *OsNRAMP5*

**Fig. 7.**
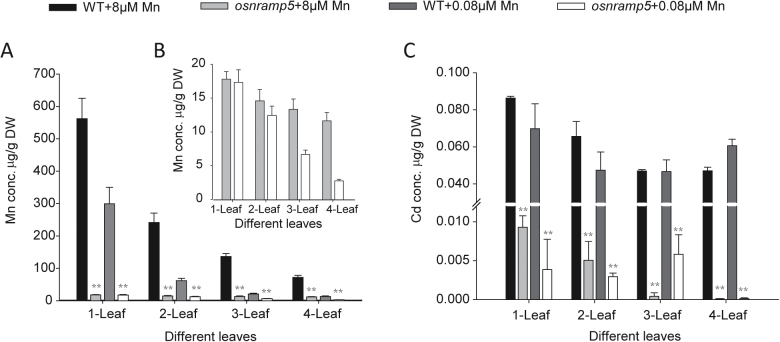
The metal concentrations in different leaves. Plants were grown under normal condition for two weeks and then shifted to different Mn supplies for additional two weeks. (A) Mn concentrations; (B) part of A; (C) Cd concentrations. Data are means±SD of three biological replicates, and five plants were mixed in one replication for metal determination. One and two asterisks indicate values are significantly different from the WT at the levels of *P*<0.05 and *P*<0.01, respectively (*t*-test).

When grown without Mn, the young leaves of *osnramp5* plants withered (data not shown). The chlorophyll concentrations of the third and fourth leaves of *osnramp5* were significantly lower than wild type under Mn-deficient conditions (Supplementary Fig. S7 available at *JXB* online). Elemental concentrations of different leaves of wild type and *osnramp5* plants grown with two levels of Mn were determined to investigate the possible role of OsNRAMP5 in Mn distribution in leaves. As expected, wild-type plants had markedly higher Mn concentrations in all leaves than the mutant (Fig. 7). Mn concentration also showed an increasing trend from young to old leaves. Unlike wild type, Mn concentrations in *osnramp5* plants differed little among different leaves at 8 µM Mn supply, whereas the third and fourth leaves contained significantly lower Mn concentrations than other leaves at 0.08 µM Mn supply (Fig. 7B). As expected, Cd in leaves was significantly lower in *osnramp5* plants compared with wild type (Fig. 7C).

### *OsNRAMP5* contributed to Mn accumulation in hulls and brown rice

*OsNRAMP5* showed strong expression in hulls (Fig. 1 and Fig. 2), suggesting that it may be involved in Mn accumulation in hulls. This hypothesis was tested by analysing the element concentrations of hulls and brown rice of the mutant and wild-type plants grown under field conditions. In wild-type plants, the concentrations of Mn, Fe, and Cd in hulls were far greater than those in brown rice (Supplementary Fig. S8 available at *JXB* online). There were significant differences in Mn concentrations between mutant and wild-type plants both in hulls and brown rice. Compared with wild-type plants, the knockout line showed an approximately 82 and 52% reduction in Mn concentrations in hulls and brown rice, respectively (Supplementary Fig. S8A available at *JXB* online). The Fe concentrations in brown rice was about 80% higher in *osnramp5* than in wild-type plants, but no significant difference was found in hulls (Supplementary Fig. S8B available at *JXB* online). Significant differences were also found in Cd concentrations of hulls and brown rice between wild type and *osnramp5* plants, with the mutant having 76% and 35% of the wild type Cd concentrations in hulls and brown rice, respectively (Supplementary Fig. S5C available at *JXB* online).

### The distributions of Mn, Fe, and Cd in root were affected by the expression pattern of *OsNRAMP5* in root

**Fig. 8.**
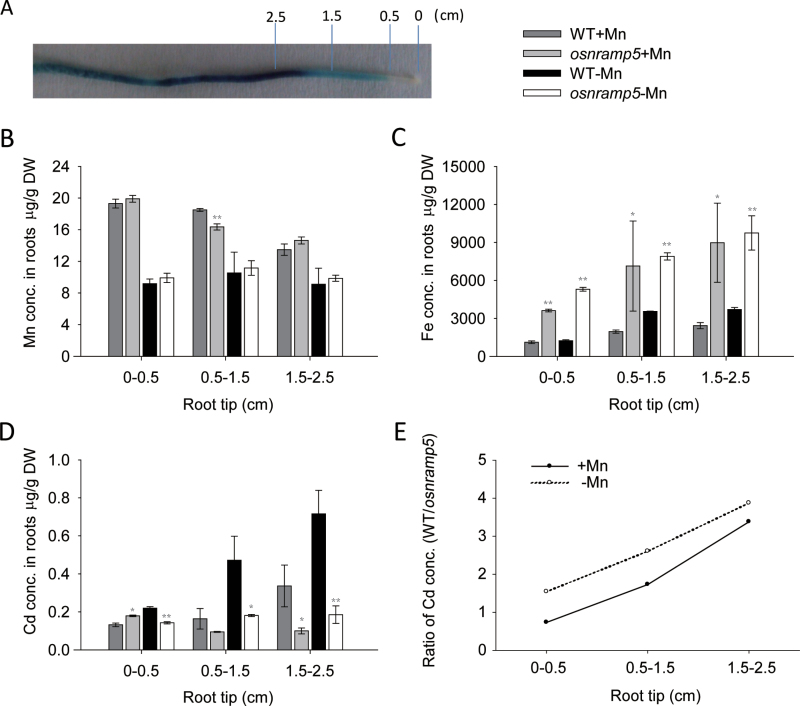
The distribution of metals in different root zones. (A) Root expression pattern of *OsNRAMP5*. The expression pattern was detected in plants transformed with the construct *OsNRAMP5*-promoter:GUS by histochemical straining of GUS activity. 0, 0.5, 1.5, 2.5 represent the distance from root tip by centimetre. (B–D) Mn, Fe, and Cd concentrations in different root zones. (E) The ratio of Cd concentrations between wild type and knock-out line of *OsNRAMP5*. Data are means±SD of three biological replicates, each sample in one replication was harvested from 30 plants. (This figure is available in colour at *JXB* online.)

In roots, the GUS activity was mainly detected in the maturation zone and, to a lesser extent, also in the elongation zone, with no activity being observed in the root cap or the meristem zone (Fig. 2). Metal concentrations in the three regions of roots were determined, showing different distribution patterns for Mn, Fe, and Cd. When grown with 8 µM Mn supply, both mutant and wild-type plants had the highest Mn concentrations in the root tip (0–0.5cm), and the concentration decreased with the distance from the apex (Fig. 8B). Under low Mn supply (0.08 µM), the difference between different root zones disappeared. Moreover, root tips of the plants grown with high Mn supply showed a two-fold increase in Mn concentration from those grown under low Mn supply. There was little difference in the Mn concentration of root tips between wild type and mutant plants. By contrast, mutant plants showed much higher Fe concentrations than wild-type plants in all three root zones (Fig. 8C). As expected, Cd concentrations of wild type were significantly higher than those of mutant plants in the root zone where *OsNRAMP5* was expressed, but not in the root tips where *OsNRAMP5* was not expressed. Moreover, the Cd concentrations of wild type increased with the distance from the root apex (Fig. 8D), which was consistent with the expression level of *OsNRAMP5* as described in Fig. 8A, but no similar pattern was observed in *osnramp5* plants. Additionally, a higher Mn supply decreased Cd concentration in the three root zones of wild type. The ratio of root Cd concentrations between wild type and mutant plants increased from the root tip to the maturation zone in both Mn treatments (Fig. 8E).

## Discussion

### The role of *OsNRAMP5* in Mn uptake and translocation

OsNRAMP5 has been reported as a major transporter for root uptake of Mn from the external medium with an apparent *K*_m_ of 1.08 µM (Sasaki *et al.*, 2012). The present study confirmed the essential role of OsNRAMP5 for Mn uptake, but also showed that OsNRAMP5 was involved in the internal translocation of Mn.

The knockout line of *OsNRAMP5* was highly sensitive to Mn deficiency (Fig. 3B). In the low range of Mn supply (0.04–1.6 µM), the Mn concentrations in shoots of wild-type plants increased markedly with increasing Mn supply, but no significant increase was found in *osnramp5* shoots. In addition, a high Mn supply could compensate for the lost capacity of Mn uptake in roots of the mutant but not the root-to-shoot translocation (Fig. 3C insert). A similar phenomenon occurred in the Fe-deficient *osnramp5* plants, which accumulated plentiful Mn in roots but did not rescue the shoot Mn concentration (Fig. 6B). The results obtained here suggest that OsNRAMP5 was involved not only in Mn uptake from the external solution but also in Mn translocation from roots to shoots. This hypothesis was also supported by the expression pattern of *OsNRAMP5* in roots: *OsNRAMP5* was expressed at high levels in stele cells especially in the xylem region, implying that OsNRAMP5 possibly participated in the xylem-mediated root-to-shoot transport (Fig. 2). In addition, Ishimaru *et al.* (2012b) found that Mn concentrations in the xylem sap from *OsNRAMP5i* plants were markedly lower than those of wild-type plants. This report also presented the expression pattern of *OsNRAMP5* in roots by the *GUS* reporter gene driven by the *OsNRAMP5* promoter, showing GUS activity around the xylem in the stele region. By contrast, Sasaki *et al.* (2012) showed that *OsNRAMP5* was specifically expressed in the exodermis and endodermis as detected by immunostaining. In the present study, *OsNRAMP5* expression was also observed in the exodermis and endodermis in the maturation zone of roots, although the level of expression was lower than that in the stele. This difference between the present study and that of Sasaki *et al.* (2012) may be caused by the different detection levels on expression pattern: *in situ* hybridization and *OsNRAMP5* promoter:β-*GUS* transgenic experiments in the present study were both at transcript level but the other study used detection at the protein level.

Although Mn is an essential microelement for plants, over-accumulation of Mn in leaves can cause severe toxicity (Pittman, 2005). Rice plants have evolved strategies to tolerate high Mn in leaves. For example, OsYSL6 is implicated in the detoxification of excess Mn (Sasaki *et al.*, 2011). However, the mechanism of Mn distribution in leaves under normal conditions of Mn supply is still poorly understood. OsYSL6 was found to be expressed in leaves with the expression level increasing with leaf age. By contrast, the present study showed that the expression of *OsNRAMP5* decreased with leaf age (Fig. 1). The higher expression of *OsNRAMP5* in the developing leaves suggests that OsNRAMP5 might play an important role in preferentially transporting Mn to young leaves. This assumption is supported by RNA *in situ* hybridization showing that *OsNRAMP5* was enriched in the vascular bundles of leaves, especially in the parenchyma cells surrounding the xylem (Fig. 2). OsNRAMP5 may therefore participate in the process of Mn unloading from the xylem vessels or in the uptake of Mn from the apoplast to the parenchyma cells. However, more direct evidence is needed. In addition, leaves of wild-type rice plants showed a pattern of Mn concentration increasing with leaf age, which was not observed in the *osnramp5* mutant (Fig. 7). This difference implies that OsNRAMP5 contributed to Mn accumulation and distribution in different leaves of rice plants.

Rice hull is important for the development of grain, and its photosynthesis contributes to grain filling (Pengelly *et al.*, 2011). It has been reported that knocking down *OsYSL2* decreased the concentrations of Mn and Fe in polished rice (Ishimaru *et al.*, 2010). The present study showed a high expression level of *OsNRAMP5* in the panicle, almost as high as that in roots, suggesting that *OsNRAMP5* may also play an important role in distributing Mn in this tissue. Both the hulls and brown rice of the *OsNRAMP5* knockout line contained much lower concentrations of Mn than wild type (Supplementary Fig. S8A available at *JXB* online), although this could be a cumulative effect of decreased root uptake, decreased root-to-shoot translocation and impaired distribution of Mn in the panicle.

### Role of *OsNRAMP5* in Fe homeostasis

There is strong evidence that Mn and Fe share some of the membrane transporters for the entrance into the root cells (Lanquar *et al.*, 2010). Indeed, Fe deficiency resulted in increased accumulation of Mn in rice roots of both the wild type and the *osnramp5* mutant, which can be explained by the induction of other Fe/Mn transporters, such as OsYSL2 (Supplementary Fig. S5 available at *JXB* online). OsNRAMP5 has been shown to possess transport activities for both Fe and Mn when expressed in yeast (Ishimaru *et al.*, 2012b). However, the total Fe concentrations in roots and shoots were hardly affected by the loss of function of OsNRAMP5. In fact, when supplied with a low level of Mn, Fe concentration of the mutant roots was increased more than that in wild type (Fig. 4A), an observation that is consistent with the results of Sasaki *et al.* (2012). This can be explained by the induction of some Fe transporters, such as *OsIRT2*, *OsYSL2*, *OsYSL15*, and *OsNRAMP1* (Supplementary Fig. S6 available at *JXB* online), in *osnramp5* plants especially under Mn-deficient conditions. That hypothesis was also supported by our data showing that a much higher Fe concentration in *osnramp5* root tips than wild type, where *OsNRAMP5* was not expressed (Fig. 8). Taken together, the results show a strong interaction between Fe and Mn and that the loss of function of OsNRAMP5 affects Fe homeostasis by influencing the expression of other Fe transporters.

### Role of *OsNRAMP5* in Cd uptake

Cd is harmful for most living organisms, including rice plants (Clemens *et al.*, 2013). OsNRAMP5 has been reported to be a major transporter for Cd uptake in rice (Ishikawa *et al.*, 2012; Sasaki *et al.*, 2012). This is supported by the results from the present study showing a marked decrease in Cd concentrations in both roots and shoots of the *osnramp5* mutant (Fig. 5). However, Ishimaru *et al.* (2012a) reported decreased Cd concentration in roots but increased Cd concentration in shoots in a knockdown line of this gene (Ishimaru *et al.*, 2012b). This inconsistency may be explained by the difference between *OsNRAMP5* knockdown and knockout lines. In the knockdown lines, Cd is taken up from soil by OsNRAMP5, and may be transported to the shoot by Fe deficiency-induced transporters.

A significant antagonism was observed between Cd and Mn in both roots and shoots in wild type, but this antagonism was not observed in the *OsNRAMP5* knockout line. In wild type, increasing Mn supply resulted in decreased Cd concentration, whereas Mn uptake was also suppressed by a high Cd supply (Fig. 4B and 5D). Moreover, the expression of *OsNRAMP5* was not affected by different Mn or Cd supply (Fig. 4G, 5G, H). The lack of Mn/Cd antagonism in the knockout mutant suggests that the antagonism observed in wild type occurs via the competition for transport by OsNRAMP5. Another possible mechanism is that high Cd and Mn were both toxic for rice plants and caused inactivation of OsNRAMP5 at the protein level to limit excessive Cd or Mn absorption.

## Supplementary data

Supplementary data are available at *JXB* online.

Figure S1. The expression pattern of *OsNRAMP5* under Mn-deficient or Fe-deficient conditions.

Figure S2. The tolerance test of wild-type and *osnramp5* to various metal deficiencies.

Figure S3. Metal concentrations in plants under different metal deficiencies.

Figure S4. The expression levels of some Fe transporters in rice roots at different Mn supplies.

Figure S5. The transcript levels of Fe transporters in rice roots under Mn and Fe deficiencies.

Figure S6. The transcript levels of Fe transporters in roots of *osnramp5* mutant and wild type plants.

Figure S7. Chlorophyll contents in different leaves of wild type and *osnramp5* mutant.

Figure S8. Metal concentrations of rice plants grown under field conditions.

Table S1. The primers of Fe transporters used in this study.

Supplementary Data
