# Supplementary material for: OsNRAMP5 contributes to manganese translocation and distribution in rice shoots
Source: J Exp Bot. 2014 Jun 24;65(17):4849–61. doi: 10.1093/jxb/eru259 (PMC4144776; doi:10.1093/jxb/eru259)
Supplement: Supplementary Data [file supp_65_17_4849__index.html]

OsNRAMP5 contributes to manganese translocation and distribution in rice shoots — OsNRAMP5 contributes to manganese translocation and distribution in rice shoots — Supplementary Data 

# OsNRAMP5 contributes to manganese translocation and distribution in rice shoots

## Supplementary Data

Data files

**Files in this Data Supplement:**

- Supplementary Data - Supplementary Data
