# Supplementary material for: OsNRAMP5 contributes to manganese translocation and distribution in rice shoots
Source: J Exp Bot. 2014 Jun 24;65(17):4849–61. doi: 10.1093/jxb/eru259 (PMC4144776; doi:10.1093/jxb/eru259)
Supplement: Supplementary Data [file supp_eru259_jexbot116566_file001.pdf]

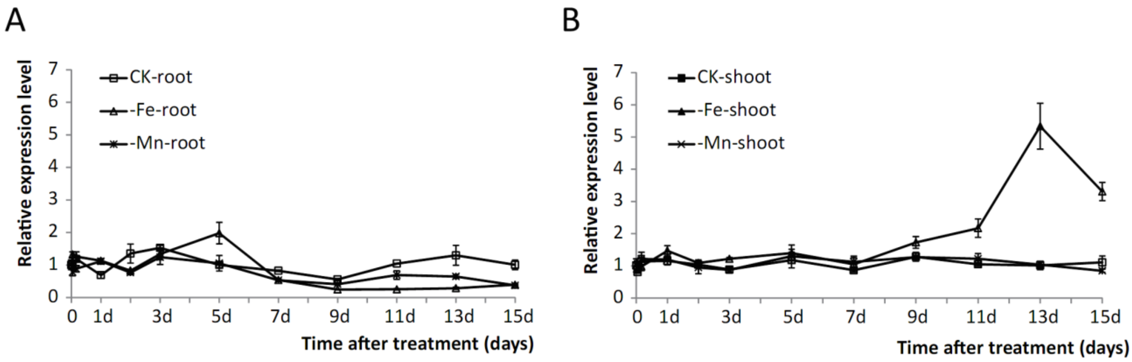

Supplementary Figure S1. The expression pattern of *OsNRAMP5* under Mn-deficient or Fe-deficient conditions. Kinetics of response of *OsNRAMP5* in both roots (A) and shoots (B) of Zhonghua 11 to Mn or Fe deficiency monitored by real-time RT-PCR. Data are means ± SD of three biological replicates.

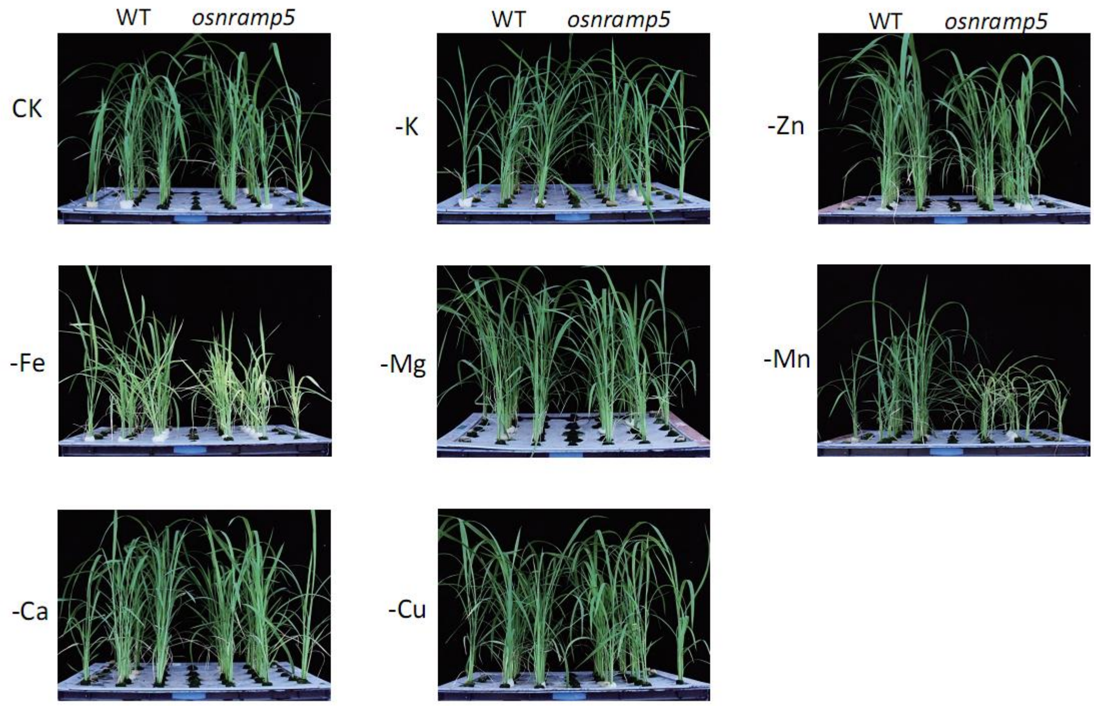

Supplementary Figure S2. The tolerance test of wild-type and *osnramp5* to various metal deficiencies. Plants are hydroponically cultivated under normal condition for 12 d and then shifted to various metal-deficient solution or continued normal solution for additional 18 d.

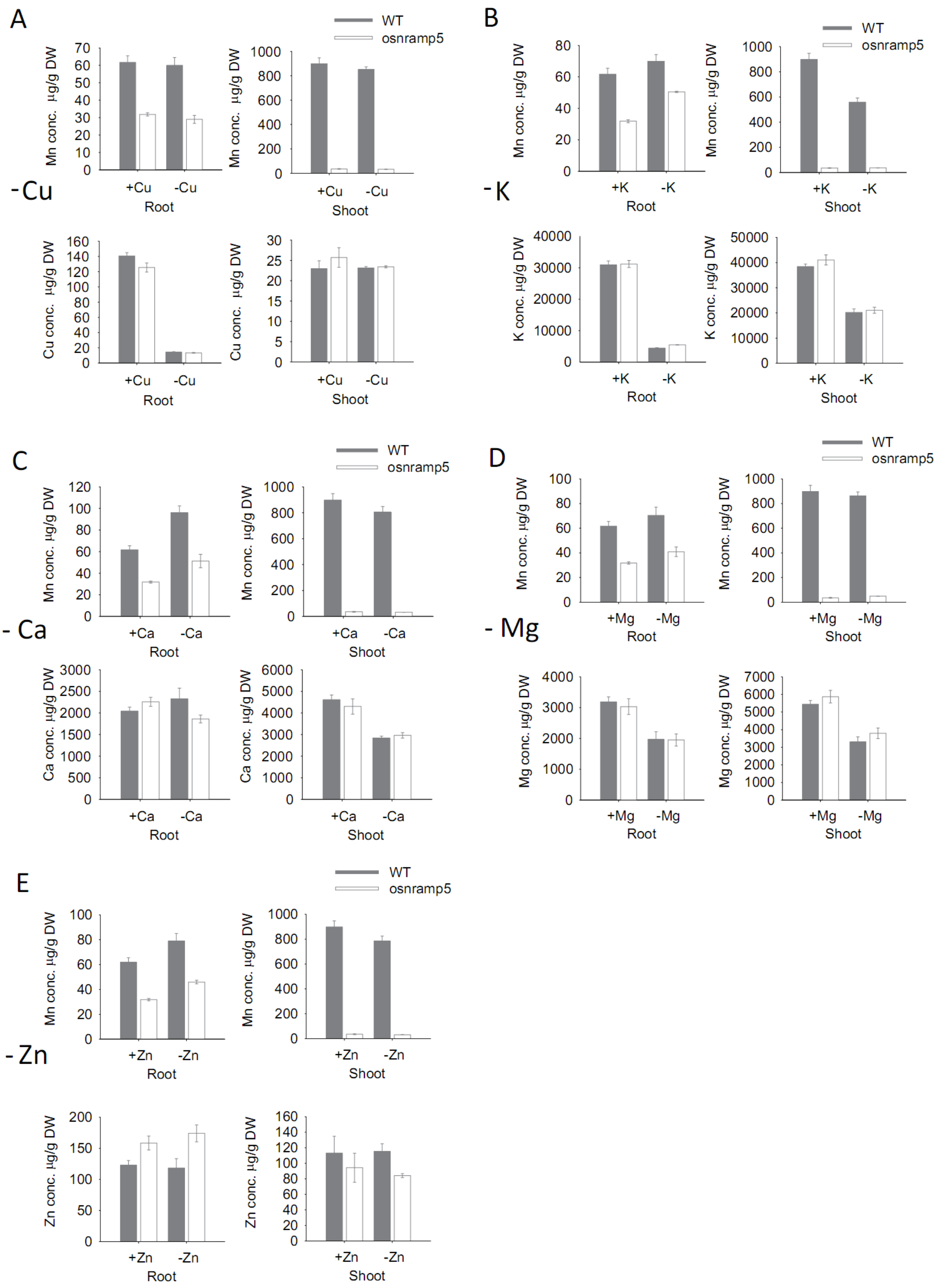

Supplementary Figure S3. Metal concentrations in plants under different metal deficiencies. Metals were analyzed in rice plants cultivated under Cu (A), K (B), Ca (C), Mg (D) and Zn (E) deficient conditions respectively. Data are means ± SD of three biological replicates, and five plants were mixed in one replication for metal determination.

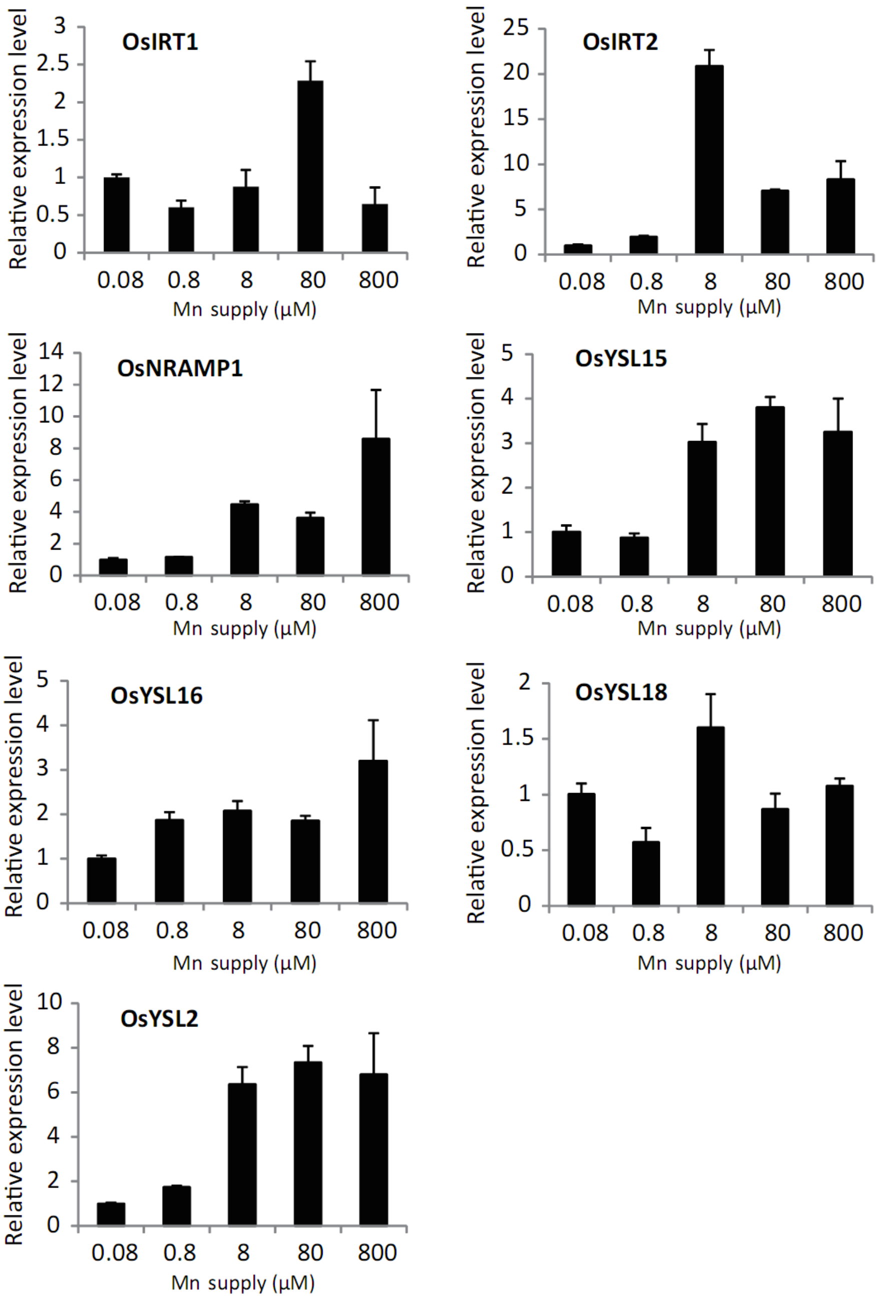

Supplementary Figure S4. The expression levels of some Fe transporters in rice roots at different Mn supplies. Plants here were cultivated under normal condition for two weeks and then shifted to different Mn supplies for additional two weeks. Data are means ± SD of three biological replicates in real-time PCR.

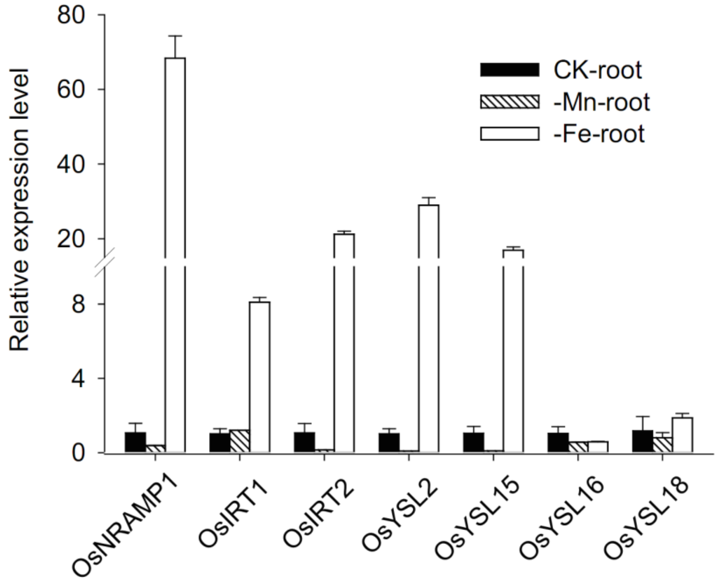

Supplementary Figure S5. The transcript levels of Fe transporters in rice roots under Mn and Fe deficiencies. Plants here were cultivated under normal condition for two weeks, and then shifted to Mn free or Fe free or continued normal condition for additional two weeks. Data are means ± SD of three biological replicates in real-time PCR.

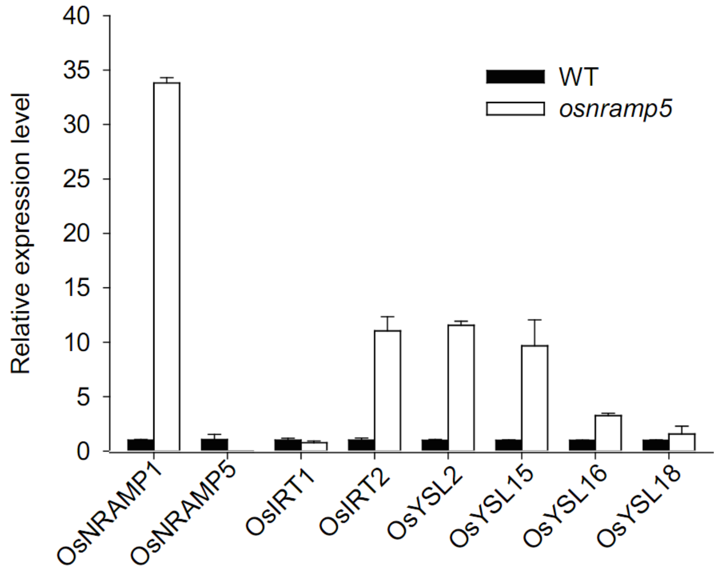

Supplementary Figure S6. The transcript levels of Fe transporters in roots of *osnramp5* mutant and wild type plants. Wild type and *osnramp5* plants here were cultivated under normal condition for four weeks. Data are means ± SD of three biological replicates in real-time PCR.

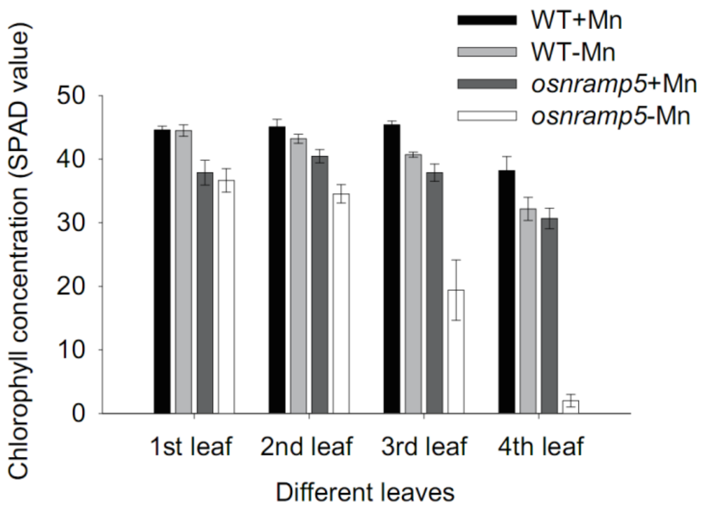

Supplementary Figure S7. Chlorophyll contents in different leaves of wild type and *osnramp5* mutant. The chlorophyll contents in different leaves of wild-type (WT) and *osnramp5* mutant plants cultivated under Mn-replete (+Mn) or Mn-free (-Mn) conditions were detected by SPAD-502. Data are means ± SD of fifteen rice plants in each sample.

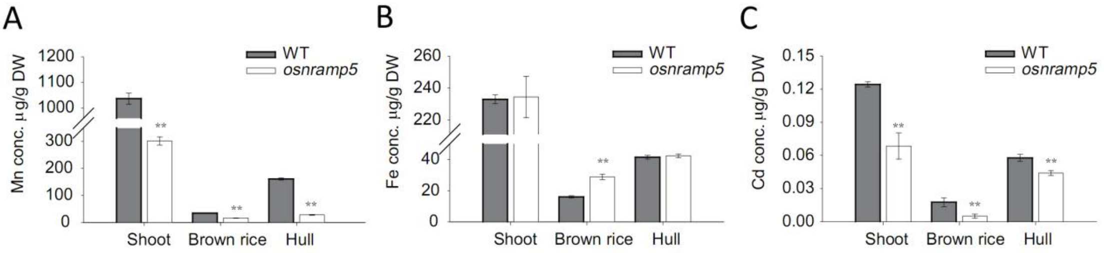

Supplementary Figure S8. Metal concentrations of rice plants grown under field conditions. Mn (A), Fe (B) and Cd (C) concentrations in rice plants cultivated under field condition were shown respectively. Data are means ± SD of three biological replicates, and five plants were mixed in one replication for metal determination. One and two asterisks indicate values are significantly different from the WT at the levels of $P<0.05$ and $P<0.01$, respectively (*t*-test).

Supplementary Table S1. The primers of Fe transporters used in this study.

Primer name	Sequence (5`-3`)
qRT-IRT1-F	GCAATTCGCTGCATTGTTAGAT
qRT-IRT1-R	ACAACATGGAGAAGTCACAGTC
qRT-IRT2-F	GCGTCAGGGTCGAAGATATG
qRT-IRT2-R	CGGCAGAAGCTGGTCTTTAT
qRT-OsNRAMP1-F	AAGGACACCGTCAAGTTCGTGTCT
qRT-OsNRAMP1-R	TAACCGGTCTGGAAGGGTACTACA
qRT-OsYSL2-F	TTCCCTTCTTCCCTGCTTTC
qRT-OsYSL2-R	CATGTCGAACTCAGCATCCA
qRT-OsYSL15-F	CTTCTATCCTTGCTCTCGCTAAG
qRT-OsYSL15-R	GCAATCCTCCACCCATGAA
qRT-OsYSL16-F	CATACCGGGAAACTAGCCAAT
qRT-OsYSL16-R	GTGTTTCTGCATTGACGCTATAC
qRT-OsYSL18-F	TCTTGGCGAGTAAGGCTAAAG
qRT-OsYSL18-R	CACTAAATAATCTACAGGTGTTGGAAG
